# Targeting SALL4 by entinostat in lung cancer

**DOI:** 10.18632/oncotarget.12251

**Published:** 2016-09-26

**Authors:** Kol Jia Yong, Ailing Li, Wen-Bin Ou, Clarice Kit Yee Hong, Wenxiu Zhao, Fei Wang, Hiro Tatetsu, Benedict Yan, Lihua Qi, Jonathan A. Fletcher, Henry Yang, Ross Soo, Daniel G. Tenen, Li Chai

**Affiliations:** ^1^ Cancer Science Institute of Singapore, National University of Singapore, Singapore; ^2^ Department of Pathology, Brigham & Women's Hospital, Harvard Medical School, Boston, MA, USA; ^3^ Zhejiang Provincial Key Laboratory of Silkworm Bioreactor and Biomedicine, College of Life Sciences, Zhejiang Sci-Tech University, Hangzhou, China; ^4^ Department of Laboratory Medicine, National University Hospital, National University Health System, Singapore; ^5^ Harvard Stem Cell Institute, Boston, MA, USA

**Keywords:** entinostat, HDAC inhibitor, lung cancer, SALL4

## Abstract

The overall survival of lung cancer patients remains dismal despite the availability of targeted therapies. Oncofetal protein SALL4 is a novel cancer target. We herein report that SALL4 was aberrantly expressed in a subset of lung cancer patients with poor survival. SALL4 silencing by RNA interference or SALL4 peptide inhibitor treatment led to impaired lung cancer cell growth. Expression profiling of SALL4-knockdown cells demonstrated that both the EGFR and IGF1R signaling pathways were affected. Connectivity Map analysis revealed the HDAC inhibitor entinostat as a potential drug in treating SALL4-expressing cancers, and this was confirmed in 17 lung cancer cell lines. In summary, we report for the first time that entinostat can target SALL4-positive lung cancer. This lays the foundation for future clinical studies evaluating the therapeutic efficacy of entinostat in SALL4-positive lung cancer patients.

## INTRODUCTION

Lung cancer is the leading cause of cancer deaths in both men and women in the United States and worldwide. It can be divided into small cell lung cancer (~20%) and non-small cell lung cancer (NSCLC, ~80%) [1]. Major treatment options for NSCLC, depending on the stages of disease, include surgery, radiation, and platinum doublet chemotherapy. In the past decade, the discovery of *EGFR* mutations and EML4-ALK fusions has led to advances in the treatment of NSCLC through the use of targeted therapies [2–4]. While other driver mutations, including *HER2*, *MET*, *NRAS*, *BRAF*, *FGFR1*, *PIK3CA*, *AKT1*, *RET*, and *ROS1* may represent viable therapeutic targets, overall they occur only at low frequency in NSCLC, with more than 50% of cases still lacking defined driver mutation [5–9]. Therefore, therapeutic options are still limited for many advanced NSCLC patients. In addition, acquired resistance to the existing targeted agents and disease recurrence present further challenges and highlight the urgent need for alternative treatment strategies [10, 11].

SALL4 is well established to be one of the critical stem cell factors for the maintenance of pluripotency and self-renewal of embryonic stem cells (ESCs) [12, 13]. Aberrant SALL4 expression has been reported in acute myeloid leukemia (AML) and a panel of solid tumors, including hepatocellular carcinoma (HCC), gastric cancer, and endometrial cancer [14–19]. Targeting SALL4 as a potential therapeutic strategy has been demonstrated in AML and HCC by interrupting the interaction between SALL4 and the histone deacetylase (HDAC) complex [15, 16]. Aberrant SALL4 expression in lung cancer patients has been reported, and the detection of SALL4 mRNA expression has been proposed as a diagnostic marker for lung cancer patients [20, 21]. However, the functional role(s) of SALL4 in NSCLC and its related mechanism, as well as its therapeutic potential in lung cancer still remain unknown. To answer these questions, we first examined the oncogenic role of aberrant SALL4 protein expression in human NSCLC. The follow-up mechanistic studies demonstrated that SALL4 affected both the EGFR and IGF1R signaling pathways by suppressing the expression of one of the E3 ubiquitin-protein ligases, CBL-B, probably through its reported interaction with the HDAC complex. Notably, our preclinical data indicates that the SALL4-expressing lung cancer cells were more sensitive to the histone deacetylase inhibitor (HDACi) entinostat (MS-275) treatment, suggesting that lung cancer patients with SALL4 overexpression may benefit from treatment with entinostat.

## RESULTS

### Aberrant SALL4 expression is detected in a subset of lung cancer and high SALL4 expression is correlated with poor survival

**Figure 1:**
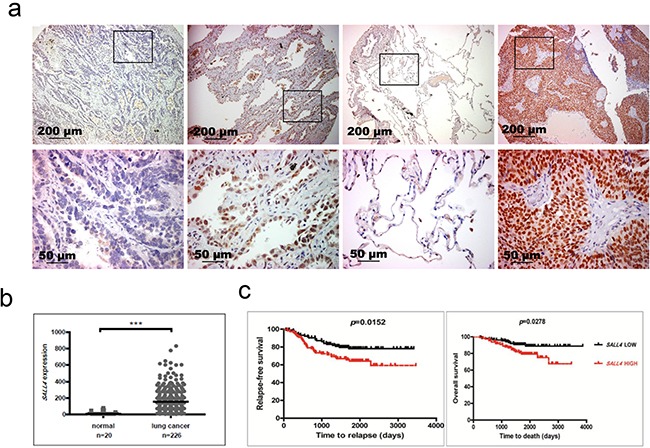
Aberrant SALL4 expression in lung cancer **a.** Representative SALL4 IHC images. Upper panel was taken with 100X magnification; lower panel was taken with 400X magnification. The left most images were from a case of adenocarcinoma with no SALL4 expression. Second left images were from a case of adenocarcinoma with +3 SALL4 expression. Second right images were the matched normal sample of the right most squamous cell carcinoma case, which has +4 SALL4 expression. The enlarged regions in the lower panel were indicated by the black rectangular boxes in the upper panel images. **b.** Gene expression profiling data analysis on 226 adenocarcinomas and 20 adjacent normal lung tissue samples (dataset GSE31210 from the GEO database) shows that *SALL4* expression is significantly higher in lung cancer samples compared to normal lung tissues (***P < 0.0001). **c.** Survival analysis demonstrates that *SALL4* expression is significantly correlated with reduced relapse-free survival and overall survival of lung cancer patients. This analysis was done on dataset GSE31210 from the GEO database.

To determine whether SALL4 is aberrantly expressed in lung cancer, we performed immunohistochemistry (IHC) to analyze the protein expression level of SALL4 in a cohort of lung cancer patients from the archives of the National University Hospital, Singapore, with normal lung tissues serving as control. Table 1 illustrates the demographic and clinicopathological characteristics of these patients. We observed elevated SALL4 expression in a subset of lung cancer patients compared to normal lung tissues (Figure 1a). Among non-small cell lung cancers (NSCLCs), 16.2% were positive for SALL4 expression. Within the NSCLC cases, SALL4 was found to be positive in 12% of adenocarcinomas (ADC) (n=100), 19% of adenocarcinoma in situ (n=21) and 23% of squamous cell carcinoma (SCC) (n=52). In addition, we evaluated RNA expression of *SALL4* in paired tumor and normal tissues from 12 lung cancer patients. Seven of these 12 lung cancer patients had increased *SALL4* expression, and overall, there was a statistically significant increase in *SALL4* expression in lung cancer tissues as compared to adjacent normal lung tissues (P=0.04) (Supplementary Figure S1).

**Table 1:** Demographic and clinicopathological characteristics of lung cancer patients from the National University Hospital, Singapore

Demographic and clinicopathological characteristics	Frequency (%)
Age (n=138)	
< median (64.33)	68 (49.3)
> median	70 (50.7)
Gender (n=139)	
Female	41 (29.5)
Male	98 (70.5)
Stage (n=127)	
1	75 (59.1)
2	25 (19.7)
3	21 (16.5)
4	6 (4.7)
Differentiation (n=123)	
Well & moderately differentiated	79 (64.2)
Poorly differentiated	44 (35.8)
Tumor size (n=128)	
< median (3.5cm)	56 (43.8)
≥ median	72 (56.2)

To validate the observation from our cohort of primary patient samples, we utilized the published expression profiling data on lung cancers (Accession GSE31210) from the Gene Expression Omnibus (GEO) database [22]. *SALL4* transcript level was analyzed in 226 adenocarcinomas and 20 adjacent normal lung tissue samples. The expression of *SALL4* was significantly increased in cancer tissues compared to normal controls (p<0.0001) (Figure 1b), confirming our observation from the immunohistochemistry staining. Using the same dataset, we further evaluated lung cancer patients with known mutations in *EGFR*, *KRAS*, and *ALK*. Lung cancer patients with *EGFR* and/or *KRAS* mutations were found to have higher *SALL4* expression, while patients with *ALK* mutations did not have significantly higher *SALL4* expression (Supplementary Figure S2). Furthermore, using the same dataset, we evaluated the prognostic value of SALL4 expression in lung cancer patients. Using median expression level as the cutoff value, we found that high *SALL4* expression in lung cancer was significantly correlated with reduced relapse-free survival and overall survival (Figure 1c), suggesting that patients with high *SALL4* expression have poorer survival advantage. Similar observation was also seen in another cohort of samples from the GEO database – elevated *SALL4* expression is observed in lung cancer patients (Accession GSE19188, Supplementary Figure S3a) [23]. Further analysis showed that there is no significant difference, in terms of *SALL4* expression level, between different subtypes of NSCLCs (Supplementary Figure S3b). To investigate whether gene sets that have prognostic values are enriched in *SALL4*-expressing lung carcinomas, we carried out Gene Set Enrichment Analysis (GSEA) by using the GSE19188 dataset. Indeed, we observed enrichment of gene sets upregulated in lung cancers with poor survival (P < 0.001) in *SALL4*-expressing group. On the other hand, gene sets that are enriched in lung cancers with good survival (P < 0.001) are enriched in the *SALL4*-negative group (Supplementary Figure S3c). These observations were confirmed by a third published primary lung adenocarcinoma dataset from the TCGA database. *SALL4* expression was significantly upregulated in adenocarcinoma tissues compared to the matched normal lung tissues (P < 0.001) (Supplementary Figure S3d). Comparing 61 samples with the highest and lowest *SALL4* expression from the same dataset, we observed significant survival advantage for the patients with low *SALL4* expression (P = 0.017) (Supplementary Figure S3d). These data collectively demonstrated that aberrant SALL4 expression is observed in NSCLCs and SALL4 has prognostic value in lung cancer.

### SALL4 expression is essential for tumorigenicity of lung cancer cells

To investigate if SALL4 has a functional role in tumorigenicity of lung cancer cells, we first screened 16 NSCLC cell lines for *SALL4* expression by using qRT-PCR in order to select for appropriate models for downstream functional studies (Supplementary Figure S4a). ADC cell line H522 and large cell carcinoma (LCC) cell line H661 showed the highest *SALL4* RNA expression level among the 16 cell lines (Supplementary Figure S4a), which was confirmed by immunoblotting at protein expression level (Supplementary Figure S4b). Moderate endogenous *SALL4* expression was detected in both H292 and PC-9 cell lines, and low or undetectable *SALL4* expression was seen in HCC827 and H1299 cells (Supplementary Figure S4a). Notably, H292 lung cancer cells have *EGFR* amplification while PC-9 lung cancer cells harbor the *EGFR* exon 19 deletion mutation.

**Figure 2:**
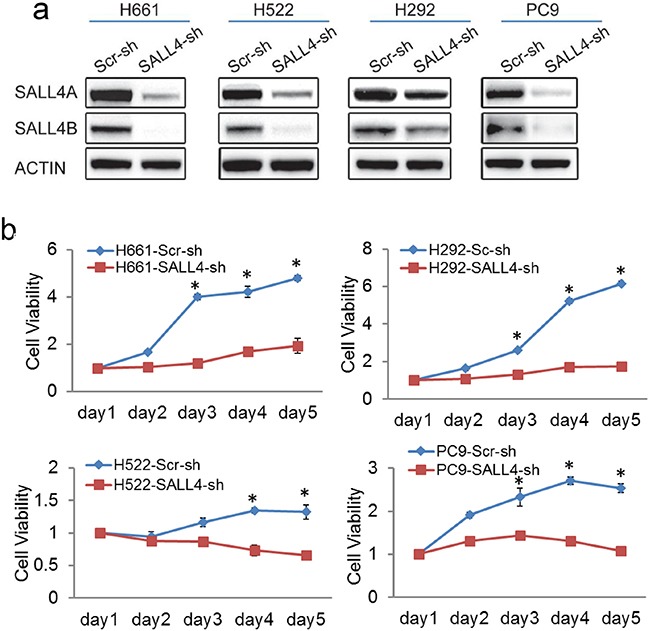

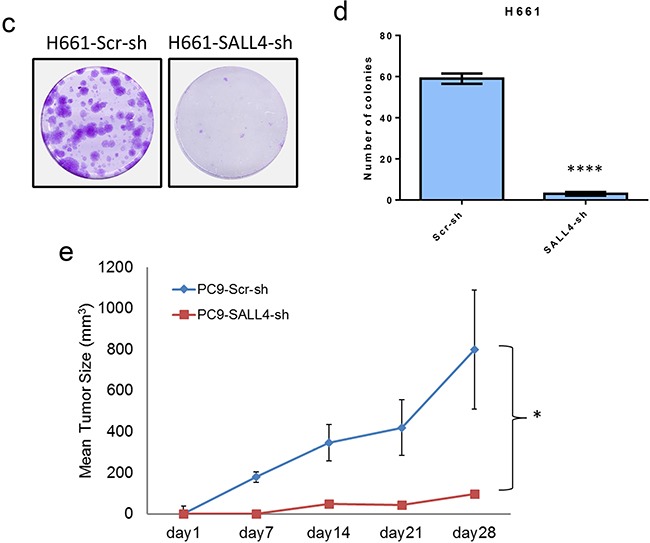
SALL4 knockdown impaired lung cancer cell viability and tumorigenicity *in vitro* and *in vivo* **a.** Western blots show SALL4 downregulation in lung cancer cell lines H661, H522, H292 and PC-9 upon lentiviral-mediated delivery of SALL4-specific shRNA. **b.** MTT assays show reduced cell viability upon SALL4 knockdown. **c.** Foci formation assay shows decreased foci formation when SALL4 is downregulated. **d.** Bar chart shows the quantitation of foci formed in both the Scr-sh and SALL4-sh groups. **** P < 0.0001, Student's t test. **e.** SALL4 depletion inhibited tumorigenecity of lung cancer cells (PC-9) in vivo. Tumor volume (mm^3^) was measured at various time points after transplantation (n = 5). Error bars indicate standard error from five mice. * P < 0.05, Student's t test. Tumor volume (mm^3^) was calculated by the following formula: (length × width× height)/2.

Next, to investigate the functional role of SALL4 in lung cancer, SALL4 expression was downregulated by lentiviral-mediated delivery of SALL4-specific short hairpin RNA (shRNA) to the high SALL4-expressing lung cancer cell lines (H661, H522, H292 and PC-9). The specificity of this SALL4 shRNA has been validated in our previous studies [15, 19]. The SALL4 shRNA transductions resulted in 50-90% inhibition of SALL4 expression at protein and mRNA levels in H661, H522, H292 and PC-9 cell lines (Figure 2a). We evaluated the effects of SALL4 knockdown on cell viability of these cells (Figure 2b). SALL4 shRNA treatment markedly reduced the number of viable cells compared to scramble shRNA control treatment (Figure 2b). Furthermore, the oncogenic role of SALL4 was investigated in an *in vitro* foci formation assay and an *in vivo* lung cancer xenograft mouse model using H661 and PC-9 cells with downregulated SALL4 expression (Figure 2c–2e). SALL4 downregulation significantly impaired the tumorigenic potential of H661 cells *in vitro* (Figures 2c and 2d). In the *in vivo* xenograft assay, shRNA-treated PC-9 cells were injected subcutaneously into NOD/SCID mice and monitored for tumor development. SALL4 knockdown dramatically inhibited tumor growth compared to scramble shRNA control group (Figure 2e). These data suggest that SALL4 expression is critical for lung tumor cell growth in vitro and in vivo.

We have recently described a SALL4 peptide as a competitive inhibitor to antagonize the oncogenic function of SALL4 in AML [15] and HCC [15, 17]. In our present study, we further evaluated the anti-proliferative effects of this 12-amino acid peptide in SALL4-expressing lung cancer cell lines. Treatment with 10 μM of TAT peptide-tagged SALL4 peptide (TAT-SALL4) led to a 30-50% reduction of cell viability in all three SALL4-positive lung cancer cell lines (H661, H522, and PC-9), while no effect was observed on non-SALL4-expressing H1299 cells, compared to control peptides (TAT, or TAT-Con) (Supplementary Figure S5). Taken together, these data suggest that SALL4 has a functional role and is a feasible drug target in lung cancer.

### Downregulation of SALL4 blocks both IGF1R and EGFR signaling pathways

**Figure 3:**
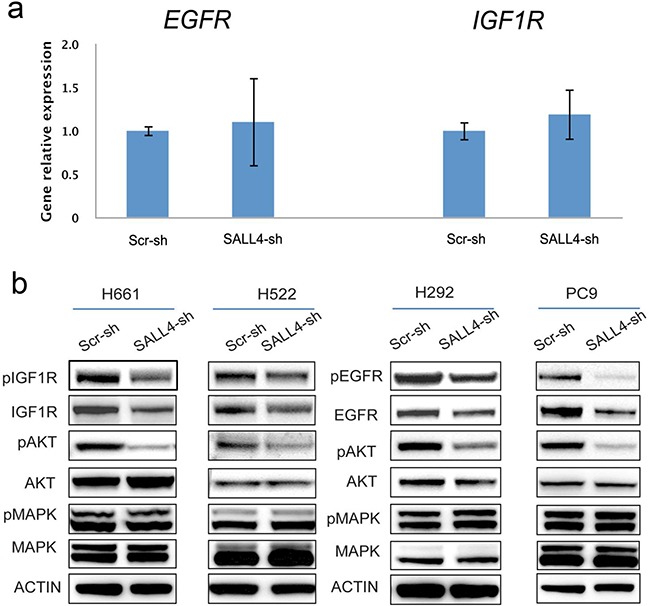
SALL4 affects EGFR and IGF1R signaling pathways indirectly **a.** qRT-PCR data shows that SALL4 knockdown in H661 cell line had no effect on the expression of EGFR and IGF1R at RNA levels, validated our microarray data. **b.** Immunoblotting shows that SALL4 knockdown inactivated IGF1R/EGFR signaling and their downstream mediator phospho-AKT, but not MAPK, in IGF1R activated H661 and H522 cell lines (left panels), and EGFR activated PC-9 and H292 cell lines (right panels), ß-actin was used as a loading control.

To elucidate the potential mechanism(s) of SALL4 in lung cancer, we performed microarray gene expression profiling on H661 cells treated with scramble shRNA control and a SALL4-specific shRNA. Combination of one-way ANOVA analysis and pathway analysis using Pathway Interaction Database (PID) showed that both EGFR (P = 1.03E-03) and IGF1R (P = 1.37E-05) signaling pathways were significantly affected by SALL4 downregulation. However, further analysis showed that SALL4 knockdown had little effects on the RNA expression levels of *IGF1R* and *EGFR* in H661 cells (Figure 3a). Nonetheless, SALL4 depletion led to decreased protein expression of total IGF1R and phospho-IGF1R in H661 and H522 cell lines (Figure 3b, left panel), as well as reduced protein expression of total EGFR and phospho-EGFR in H292 and PC-9 cells (Figure 3b, right panel), despite the unchanged RNA expression levels. Furthermore, SALL4 knockdown also inactivated the IGF1R/EGFR downstream intermediate target AKT, as seen from downregulation of phospho-AKT, but not MAPK in the four lung cancer cell lines examined (Figure 3b). These data suggest that depletion of SALL4 expression in lung cancer cells blocks both the EGFR and IGF1R signaling pathways, albeit not through direct regulation of *EGFR* and *IGF1R* mRNA expression.

### SALL4 regulates EGFR and IGF1R signaling pathways through CBL-B ubiquitin ligase

**Figure 4:**
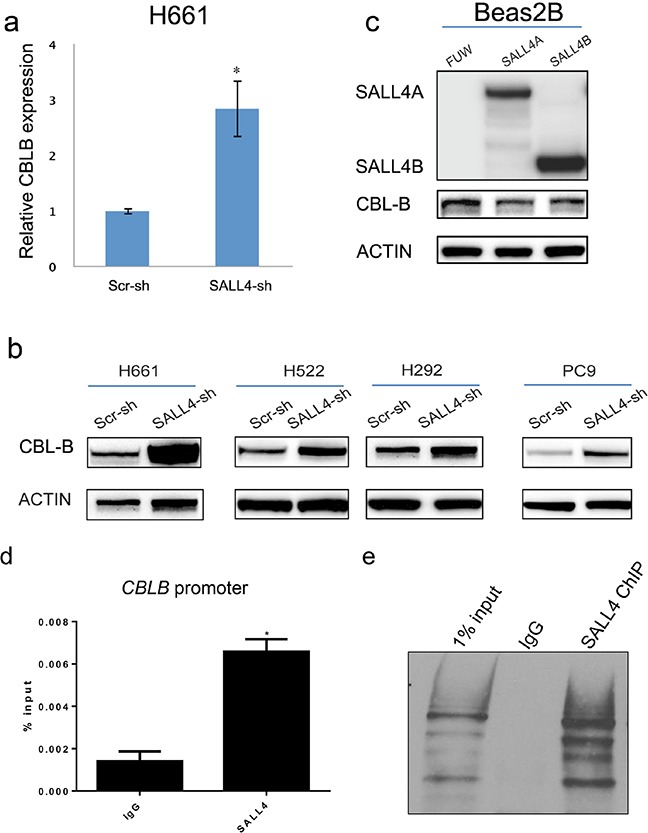
SALL4 represses CBL-B, an E3 ubiquitin ligase implicated in the degradation of EGFR and IGF1R **a.** qRT-PCR data shows that SALL4 knockdown in H661 cell line induced CBLB expression at RNA levels, confirming our microarray data. **b.** SALL4 knockdown resulted in upregulation of CBL-B in four lung cancer cell lines at protein levels. **c.** Ectopic overexpression of SALL4 significantly decreased CBL-B expression in a normal lung cell line (Beas2B). **d.** ChIP-qPCR experiments indicate that SALL4 binds to the *CBLB* promoter in H661 cells. * P < 0.05, Student's t test. **e.** ChIP-WB shows enrichment of SALL4 protein in SALL4 ChIP sample.

It is known that receptor tyrosine kinases (RTKs), including EGFR and IGF1R, can be negatively regulated by CBL RING ubiquitin ligases [24, 25]. Interestingly, our microarray data showed that loss of SALL4 led to an upregulation of *CBLB*, a member of the CBL RING ubiquitin ligases, which was further confirmed by qRT-PCR (Figure 4a). In addition, SALL4 knockdown induced CBL-B protein expression in four lung cancer cell lines examined (H661, H522, PC-9, and H292) (Figure 4b). The effects of SALL4 on CBL-B expression were further evaluated by western blotting after overexpression of SALL4 in a normal lung epithelial cell line (Beas2B). Ectopic expression of both SALL4 isoforms A and B suppressed CBL-B expression (Figure 4c). These results suggest that SALL4 augments EGFR and IGF1R expression in lung cancer through the downregulation of CBL-B ubiquitin ligase.

We next performed SALL4 chromatin immunoprecipitation (ChIP)-qPCR to investigate if SALL4 binds directly on the promoter region of *CBLB*, thereby repressing its expression. Significant enrichment of SALL4 binding on *CBLB* promoter was observed, as compared to the isotype control (Figure 4d), suggesting that SALL4 might regulate the expression of CBL-B directly by transcription repression. Figure 4e shows the enrichment of SALL4 protein in our ChIP sample.

### Integrated analysis using connectivity Map reveals that HDAC inhibitors can reverse SALL4 gene expression signature in lung cancer

While we have identified a peptide to specifically inhibit the growth of SALL4-expressing lung cancer cells, the clinical utility of peptides, in general, is limited by their sub-optimal in vivo delivery efficiency. To search for potential existing drug(s) to treat SALL4-expressing cancers, we conducted Connectivity Map (cMap) analysis [26, 27]. The SALL4 gene signature obtained from either our knockdown experiments or from data mining on primary lung cancer patients was compared to the drug signatures through the cMap site (http://www.broadinstitute.org/cmap/) with over thousands of expression profiling results upon drug treatments. The outcome of comparison was given a score from +1 (maximum positive correlation) to -1 (maximum negative correlation) based on the extent of correlation between the two gene signatures. In our case, the drugs that received a score close to -1 are most likely to have therapeutic values for the SALL4-expressing lung cancer cells since the drug signatures are in a reverse correlation with the SALL4 gene signature.

We first performed cMap analysis using one set of SALL4 gene signature generated from the comparison between normal human control versus primary lung cancer samples (Supplementary Tables S1 and S2). A separate SALL4 gene signature generated from differentially expressed genes following SALL4 knockdown in the lung cancer cell line H661 (Supplementary Table S3) was also used by cMap. Comparative analysis identified overlapping of three candidate drugs in all three analyses using the various gene signatures (Supplementary Table S4). Intriguingly, entinostat (MS-275), a HDACi, was among the three common drugs. This, however, was not unexpected, as we have reported in our previous studies that one of the main functions of SALL4 is to act as a transcription repressor by interacting with the nucleosome remodeling and deacetylase (NuRD) complex that comprises of HDAC1 and HDAC2 [28]. As an HDACi, entinostat preferentially inhibits HDAC1 as compared to HDAC3 [29], and has been used in clinical trials to treat lung cancer patients [30].

### SALL4-expressing lung cancers are sensitive to HDAC inhibitor entinostat treatment

**Figure 5:**
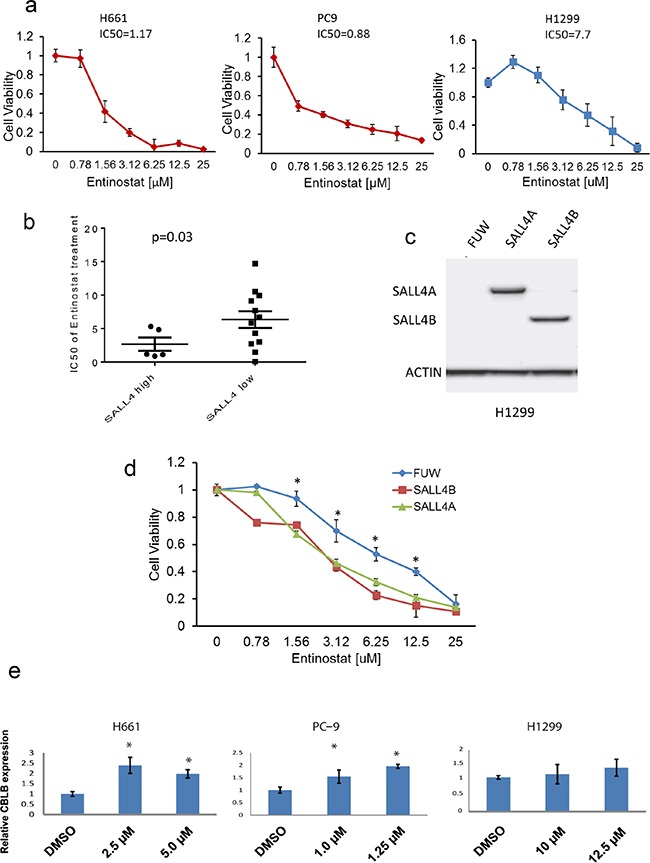

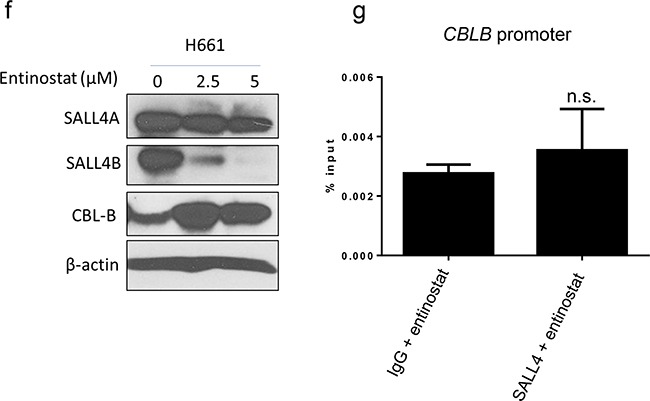
SALL4-expressing cells are more sensitive to HDACi entinostat treatment **a.** MTT assay shows the inhibition of cell growth in lung cancer lines (H661, PC-9 and H1299) after 72 h treatment with HDACi entinostat at various dosage (0.78, 1.56, 3.12, 6.25, 12.5, and 25 μM). Data were normalized to the DMSO control, and represent the mean values (± s.d.) of quadruplicate cultures from two independent experiments. **b.** IC_50_ values of entinostat treatment was plotted against two groups of lung cancer cell lines (n = 17) with high or low *SALL4* expression. Entinostat sensitivity is correlated positively with *SALL4* expression. **c.** Western blot shows overexpression of SALL4 isoforms A and B in the SALL4 negative lung cancer cell line H1299 **d.** MTT assay shows increased sensitivity of H1299 cells to entinostat treatment upon SALL4 overexpression when compare to FUW empty vector control (* P < 0.05). **e.** qRT-PCR shows *CBLB* expression in H661, PC-9, and H1299 cells after treatment with entinostat for 48 h. **f.** Western blot shows reduced SALL4 expression and increased CBL-B expression in H661 cells after entinostat treatment for 72 h. **g.** ChIP-qPCR shows decreased SALL4 binding to the *CBLB* promoter upon treatment with 10μM entinostat for 24h. n.s.: not significant

To validate the cMap result, we conducted further studies using the HDACi entinostat. A panel of 17 lung cancer cell lines with different expression levels of SALL4 was evaluated for their drug sensitivity to entinostat. We first noticed that lung cancer cells with high SALL4 expression levels such as H661 and PC-9 cells were more sensitive to entinostat treatment than those with low SALL4 expression level, such as H1299 cells. The IC_50_ values of entinostat at day 3 were 1.17 μM for H661, 0.88 μM for PC-9, and 7.7 μM for H1299, respectively (Figure 5a). Furthermore, based on SALL4 mRNA expression level, 17 lung cancer cell lines were stratified into “SALL4 high” and “SALL4 low” groups using the mean *SALL4* expression level as threshold. The entinostat sensitivity was then compared between these two groups (Figure 5b). A negative correlation between the IC_50_ of entinostat and SALL4 expression level was observed, suggesting that lung cancer cells with high SALL4 expression were more sensitive to entinostat treatment (P = 0.03) (Figure 5b). To further evaluate the correlation between SALL4 expression and entinostat treatment sensitivity, SALL4 isoforms A and B were overexpressed in SALL4 negative lung cancer cell line H1299 (Figure 5c). Ectopic expression of SALL4 in these cells led to increased sensitivity of the cells to entinostat treatment, as indicated by a reduced IC_50_ value (Figure 5d).

Since SALL4 can repress its downstream target gene expression through its interaction with the HDAC-containing complex NuRD, we reason that entinostat, an HDACi, can potentially affect the repressive function of SALL4. We have shown in this study that SALL4 can repress CBL-B expression in lung cancer cells. We next examined whether the expression of CBL-B in these cells was affected upon entinostat treatment. The expression levels of CBL-B were increased significantly only in SALL4-expressing H661 and PC-9 cell lines and not in SALL4-non-expressing H1299 cells after entinostat treatment (Figure 5e). Additionally, entinostat treatment led to a decrease in SALL4 expression levels (Figure 5f), and abolished the enrichment of SALL4 binding on *CBLB* promoter as observed from SALL4 ChIP-qPCR in H661 cells (Figure 5g).

To our knowledge, these data demonstrate for the first time that high SALL4 expression is associated with a more sensitive response to entinostat treatment in human lung cancer cells. Therefore, SALL4 might be a good predictive marker for entinostat, and possibly other HDAC inhibitors in lung cancer.

## DISCUSSION

SALL4 is an important stem cell factor [12, 13], and it has recently emerged as an oncofetal protein [15]. Aberrant SALL4 expression has been reported in a myriad of solid tumors including HCC [15, 16, 31], endometrial cancer [19], ovarian cancer [32], breast cancer [33], gastric cancer [18, 34], lung cancer [20, 21], and germ cell tumors [35–37], as well as in leukemias [14, 38, 39]. Its druggability has been proposed in liver cancer and AML [15]. Lung cancer is the leading cause of cancer deaths in the United States (US) and worldwide. Recently, molecular targeted therapies including EGFR and ALK inhibitors have resulted in some improvement in the treatment response in selected groups of NSCLC patients. However, EGFR and ALK aberrations collectively account for only approximately 15% of all NSCLCs in the US, and the overall survival rate for lung cancer patients remains extremely poor (5-year survival rate of ~15%). Therefore, more effective therapeutics are needed for lung cancer patients, particularly for the ones that are negative for EGFR mutations.

In our present study, we first examined SALL4 expression in a cohort of primary lung cancer samples using immunohistochemical staining. 16% of 173 primary lung cancer patients demonstrated positive SALL4 expression, whereas no expression of SALL4 was observed in normal lung tissues (except for the basal layer of bronchiolar epithelium), consistent with published results by other groups [37, 40, 41]. The observation that SALL4 is overexpressed in a subgroup of lung cancers was further validated by analysis of published gene expression profiles of lung cancers in public databases. Importantly, we have also observed positive SALL4 expression in EGFR mutation negative lung cancer patients. The correlation between SALL4 expression and poorer patient survival in lung cancer is consistent with our observations from HCC, endometrial cancer and myelodysplastic syndrome. The unique expression pattern of SALL4 in lung cancer suggests that it can potentially be a good drug target for personalized medicine, targeting the lung cancer subtypes that cannot be treated by current targeted therapies like EGFR and ALK inhibitors.

To date, targeted therapies have reshaped the management of various types of cancers including NSCLC. EGFR is frequently strongly activated and overexpressed in NSCLC. Oncogenic *EGFR* mutation is a compelling therapeutic target in NSCLCs and numerous agents have been developed to target this moiety [42–44]. However, cancer cells may circumvent the drug effects of EGFR inhibitors by activating alternative pathways, thus limiting the efficacy of such therapeutic approaches [45, 46]. One of these alternative pathways is the IGF1R pathway, which is an important cell survival pathway. In the setting of NSCLC, increased expression of IGF1R appears to be detected in up to 70% of patients [47]. Also IGF1R overexpression was found to correlate with decreased efficacy of EGFR targeting, suggesting the importance of IGF1R signaling in EGFR inhibitors drug resistance [48, 49]. All these studies suggest that co-targeting of both IGF1R and EGFR represent a more viable therapeutic option for NSCLC.

In this study, we show that depletion of SALL4 by shRNA resulted in a decreased in cell viability and tumorigenicity of SALL4-positive lung cancer cells, which were associated with dysregulation of both EGFR and IGF1R signaling pathways. The regulatory effects of SALL4 on these two pathways seem to be indirect as SALL4 had no direct effect on the mRNA expression of *EGFR/IGF1R*. Instead, SALL4 silencing induced the expression of CBL-B, an E3 ubiquitin ligase, both at the protein and mRNA levels. Members of the CBL protein family (CBL, CBL-B and CBL-C) have emerged as dominant “activated RTK-selective” ubiquitin ligases, which serve a unique role as activation-induced feedback negative regulators of RTKs, including EGFR, PDGFR, MET, KIT, and IGF1R. Various studies have demonstrated that CBL protein-dependent ubiquitination targets activated RTKs for degradation either by promoting their proteasomal degradation or by facilitating their endocytic sorting into lysosomes. Data from lung cancer cell lines suggest that SALL4 downregulation may result in degradation of EGFR and IGF1R proteins, possibly in part through CBL-B. This model needs to be verified further by rescue experiments in the future.

We have previously identified a peptide that can block the oncogenic function of SALL4 by interrupting the interaction between SALL4 and the NuRD complex [15]. While the development of SALL4-specific inhibitor(s) that mimic this SALL4 peptide is ongoing, we set out to search for existing drugs that can potentially treat SALL4-expressing cancers. Using cMap predication, we identified that the drug signature from HDAC inhibitor entinostat significantly correlates with SALL4 gene signature. Using a panel of 17 lung cancer cell lines with varying SALL4 expression levels as a preclinical model, we further confirmed that high SALL4 expression lung cancer cells were more sensitive to entinostat treatment. Entinostat can induce the expression of *CBLB*, a gene that is repressed by SALL4 in lung cancer cells, representing a potential mechanism for the sensitivity of SALL4-expressing lung cancer cells to this drug. entinostat is an HDACi targeting class I enzymes HDAC1 to HDAC3. Recent clinical trials conducted on patients with lung cancer reveal that combination of entinostat with existing lung cancer treatments, such as erlotinib or azacitidine, appears promising, particularly for the carefully selected subgroups of patients [30]. Currently, there is no predictive biomarker for entinostat for lung cancer treatment. Our preclinical studies using a panel of 17 lung cancer cell lines suggest that SALL4 could be a useful predictive marker for entinostat. Future clinical studies are needed to investigate whether SALL4 can be used as a biomarker in selecting lung cancer patients who may benefit from combination therapy with entinostat.

In summary, this is the first comprehensive study on the role of SALL4 in lung cancer. We have evaluated SALL4 expression in lung cancer in large cohorts of patients, and observed that SALL4 expression is upregulated in a subset (about 16%) of these patients including EGFR mutation negative cases. As reported in other types of cancers, SALL4 plays a critical role in lung cancer cell survival. This is supported by genetic (SALL4 knockdown) and pharmacological (peptide) studies. SALL4-expressing lung cancer cells are more sensitive to entinostat treatment. Overall, our studies suggest that targeting SALL4 by entinostat is a novel approach for lung cancer treatment.

## MATERIALS AND METHODS

### Patient samples

173 cases of primary lung cancer tissues (52 SCC, 100 ADC, and 21 BAC) and 166 cases of matched normal lung tissues from the archives of the National University Hospital, Singapore were included in this study. Ethics approval was obtained from the National University of Singapore Institutional Review Board (NUS IRB 09-261).

### Antibodies and reagents

All phospho-antibodies (pIGF1R #3024, pEGFR #2234, pAKT #9271, pMAPK #9101) and polyclonal antibodies to AKT (#9272) and IGF1R (#3027) were from Cell Signaling Technology (Beverly, MA). Monoclonal mouse antibodies to SALL4 (#sc-101147) and CBL-B (#sc-1705), and polyclonal antibody to EGFR (#sc-03) were from Santa Cruz Biotechnology (Santa Cruz, CA). Polyclonal antibody to MAPK (#61-7400) was from Invitrogen Life Technologies (Carlsbad, CA). HDAC inhibitors (trichostatin A and entinostat) were provided by Dr. James Bradner's lab at DFCI.

### Cell lines

Lung tumor cell lines (PC-9, H460, A549, H1299, H520, A427, H292, H441, H322, H1650, H1395, HCC827, H3122, H1975, H3255, and H23) and a normal lung epithelial cell line (Beas2B) were kindly provided by Drs. Daniel G. Tenen and Susumu Kobayashi. H522 and H661 cell lines were purchased from the American Type Culture Collection (ATCC).

### SALL4 immunohistochemistry

IHC staining was performed according to the standard techniques as described previously [15].

### Lentiviral SALL4 overexpression and *SALL4* shRNA constructs and lentivirus packaging

Human SALL4A or SALL4B fragments were subcloned into a modified lentiviral vector (FUW-Luc-mCh-puro) with mCherry and puromycin selection markers. Lentivirus preparations were produced by cotransfecting empty vector pLKO.1puro with *SALL4 shRNA* or FUW with *SALL4* ORF, and helper virus packaging plasmids pCMVΔR8.91 and pMD.G (at a 10:10:1 ratio) into 293T cells. Transfections were carried out using lipofectamine and PLUS reagent. Lentiviruses were harvested at 24, 36, 48, and 60 hrs post-transfection, and frozen at −80°C in aliquots at appropriate amounts for infection. Validated shRNAs were used for SALL4 knockdowns. The sequences for *SALL4 shRNA* and scrambled shRNA are as follows: *SALL4* shRNA: 5′-GCCTTGAAACAAGCCAAGCTA-3′; and Scr-shRNA: 5′-CCTAAGGTTAAGTCGCCCTCG-3′.

### Cell culture and virus infection

H661, H522, PC-9, H292 and H1299 cell lines were maintained in RPMI1640 medium plus 15% fetal bovine serum (FBS) containing penicillin/streptomycin and L-glutamine. Beas2B was maintained in F10 containing 10% FBS, penicillin/streptomycin, L-glutamine, amphotericin, Mitotracker+, and bovine pituitary extract. Transduction was carried out as described previously^48^.

### Cell viability analysis

Lung cancer cells were plated at 5,000 cells/well in a 96-well flat-bottomed plate (Falcon, Lincoln NJ) and cultured in RPMI1640 for 24 hours before transduction with lentiviral empty vector, *SALL4* shRNA, or treatment with HDAC inhibitors (entinostat or TSA), or TAT-SALL4 peptide (YGRKKRRQRRRMSRRKQAKPQHI)/TAT-con peptide(YGRKKRRQRRR). Cell viability was assessed 1-5 days after transduction or inhibitor treatment using the MTT assay as described^14, 22, 48^. Data were normalized to the empty vector group or DMSO. All assays were performed in quadruplicate wells, and were averaged from two independent experiments for each cell line.

### Foci formation assay

Lung cancer cells (H661) were treated with scrambled shRNA (Scr-sh) or SALL4 shRNA (SALL4-sh). Three days after lentiviral transduction, cells were plated at 500 cells per well in a 6-well plate (Corning, NY) and cultured in RPMI1640 medium for 2 weeks. Colony formation was observed by staining with crystal violet solution (0.1% crystal violet/ 25% methanol) and the number of colonies was manually counted. All assays were performed in triplicate wells and averaged from two independent experiments.

### Xenotransplant murine models

All experimental procedures involving animals were conducted in accordance with the institutional guidelines set forth by the Children's Hospital Boston (CHB animal protocol number 11-09-2022). Eight to 10 week-old NOD/SCID mice were housed in a specific pathogen-free facility. Athymic nude mice were injected subcutaneously with PC-9 cells expressing SALL4-targeting shRNA on one flank (n=3 each) and scramble shRNA control on the other flank (n=3). 2 × 10^6^ infected cells on puromycin selection were resuspended in BD Matrigel and implanted subcutaneously at each injection site. After injection, mice were examined and tumor volumes were measured at various time points. Tumor volume was calculated by using the formula, tumor volume = (length × width× height)/2. Mice were killed by CO_2_ inhalation and necropsied 4 weeks after injection, and then tumor volume was evaluated.

### Chromatin Immunoprecipitation (ChIP)-qPCR

H661 cells were cross-linked with 1% formaldehyde and snap-frozen until processed. 10 million cells were used per experiment. Cells were lysed in 1ml of cell lysis buffer (20mM Tris, pH8/ 85mM KCl/ 0.5% NP-40 and 1X cOmplete protease inhibitor cocktail), and the nuclei was resuspended in nuclear lysis buffer (10mM Tris-HCl, pH7.5/ 1% NP-40/ 0.5% NaDOC/ 0.1% SDS/ 1X cOmplete protease inhibitor cocktail). The nuclei were then sonicated using the Biorupter (30s on/off pulses for 10 cycles). The salt concentration of the sheared chromatin was adjusted to 500mM NaCl. Immunoprecipitations were then carried out with 1.9μg of SALL4 antibody in ChIP dilution buffer (16.7mM Tris-HCl, pH8.1/ 500mM NaCl/ 0.01% SDS/ 1.1% Triton X-100/ 1.2mM EDTA) overnight. Bead complexes were washed twice with RIPA buffer high salt (500mM), then twice with LiCl buffer (10mM Tris-HCl, pH8.1/ 1mM EDTA/ 1% NaDOC/ 1% NP-40/ 250mM LiCl), and finally twice in TE buffer. Protein-DNA complexes were then eluted with 100μl of ChIP elution buffer (10mM Tris-HCl, pH8/ 5mM EDTA/ 300mM NaCl/ 0.1% SDS) and 16μl of reverse-crosslinking buffer (250mM Tris-HCl, pH6.4/ 1.25M NaCl/ 62.5mM EDTA/ 5mg/ml Proteinase k/ 62.5mg/ml RNAse A) by incubated at 65°C overnight. DNA samples were then PCR purified with QIAquick PCR Purification Kit (Qiagen, Germany) and analysed by qPCR. ChIP-qPCR primers used were: CBLB promoter forward: 5′- CAGCATCAATAATACCCAAAATTCGACC-3′, reverse: 5′- CGATGGAGGAAGATGCAGTGGTAC-3′.

### Primers

The qRT-PCR assays for *SALL4, EGFR, IGF1R and CBLB* were performed using the following primers: *SALL4* sense: 5′-CCGTCCATGCGGAAGATC-3′ and anti-sense: 5′-GAAGACCTCCTCCTCGCACT-3′; *EGFR* sense: 5′-CCCCCAGCGTATCTATATGGAA-3′ and anti-sense: 5′-GCTGTCCCTCTCCACTGCAA-3′; ^5^ *IGF1R* sense: 5′-GAGAGCTGGTAGTTAGTAGCATGTTGA-3′ and anti-sense: 5′- AATTCCAATAATGAACCCAATAGATTAGTTA -3′^17^. *CBLB* sense: 5′-GAGAGCTGGTAGTTAGTAGCATGTTGA-3′ and anti-sense: 5′- AATTCCAATAATGA ACCCAATAGATTAGTTA -3′. As controls, *GAPDH* was amplified using the following primers: *GAPDH* sense: 5′-GAAGGTGAAGGTCGGAGTCAAC-3′ and anti-sense: 5′-TGGAAGATGGTGATGGGATTTC-3′. All primers were obtained from Invitrogen. The comparative C_t_ (cycle threshold) method was used to determine RNA expression fold differences in lung cancer cell lines. The data points (run in triplicate assays) were normalized to *GAPDH*.

### Microarray

Total RNA was extracted using Qiagen RNeasy Mini Kit (Qiagen, Hilden, Germany) following manufacturer's instructions. RNA quantity was assessed using Nanodrop (Thermo Fisher Scientific, Wilmington, DE) and RNA integrity was analyzed using Nano chip for Eukaryotes on the Agilent 2100 Bioanalyzer (Agilent Technologies, Santa Clara, CA). A RIN value of 8.0 and above was considered to indicate a satisfactory sample quality. Gene expression array was carried out as described previously^48^. The microarray data was deposited in the GEO database with the Accession number GSE68959.

### GSEA analysis

Gene expression data of lung cancers was extracted from the GEO database (accession number: GSE31210 and GSE19188). Before GSEA, all lung cancer samples were divided into two groups according to their SALL4 expression levels: SALL4 high and SALL4 low. GSEA was then performed based on normalized data using GSEA v2.0 tool (http://www.broad.mit.edu/gsea/) for identification of enriched gene sets between the SALL4 high and SALL4 low groups.

### Statistical analysis

Results are expressed as mean ± s.d. from at least three independent experiments, unless otherwise stated. Statistical significance between the two groups was determined by Student's t-test or Pearson's Chi-squared (χ2) test (GraphPad Prism 5.0 software (La Jolla, CA, USA). Log-rank test was used for survival analysis. The value of P < 0.05 was considered statistically significant and is indicated by an asterisk.

## SUPPLEMENTARY FIGURES AND TABLES








