# Supplementary material for: Targeting SALL4 by entinostat in lung cancer
Source: Oncotarget. 2016 Sep 26;7(46):75425–40. doi: 10.18632/oncotarget.12251 (PMC5342750; doi:10.18632/oncotarget.12251)
Supplement: Supplementary file 1 [file oncotarget-07-75425-s001.pdf]

Targeting SALL4 by entinostat in lung cancer

SUPPLEMENTARY FIGURES AND TABLES

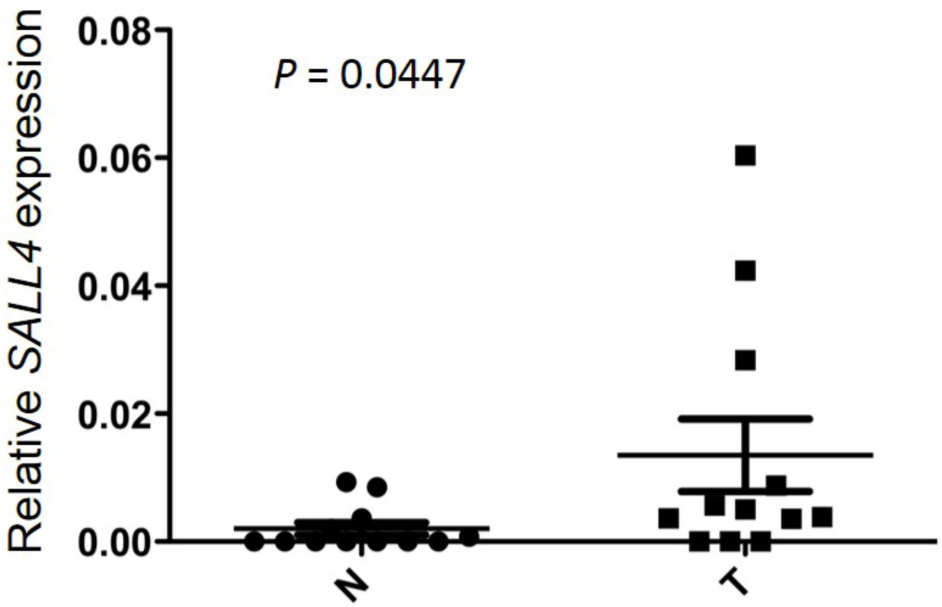

Supplementary Figure S1: *SALL4* mRNA expression is upregulated in lung cancer patients. qPCR data demonstrates significantly upregulated *SALL4* expression in 12 primary lung cancer tissues analyzed, as compared to the matched non-tumor lung tissues.

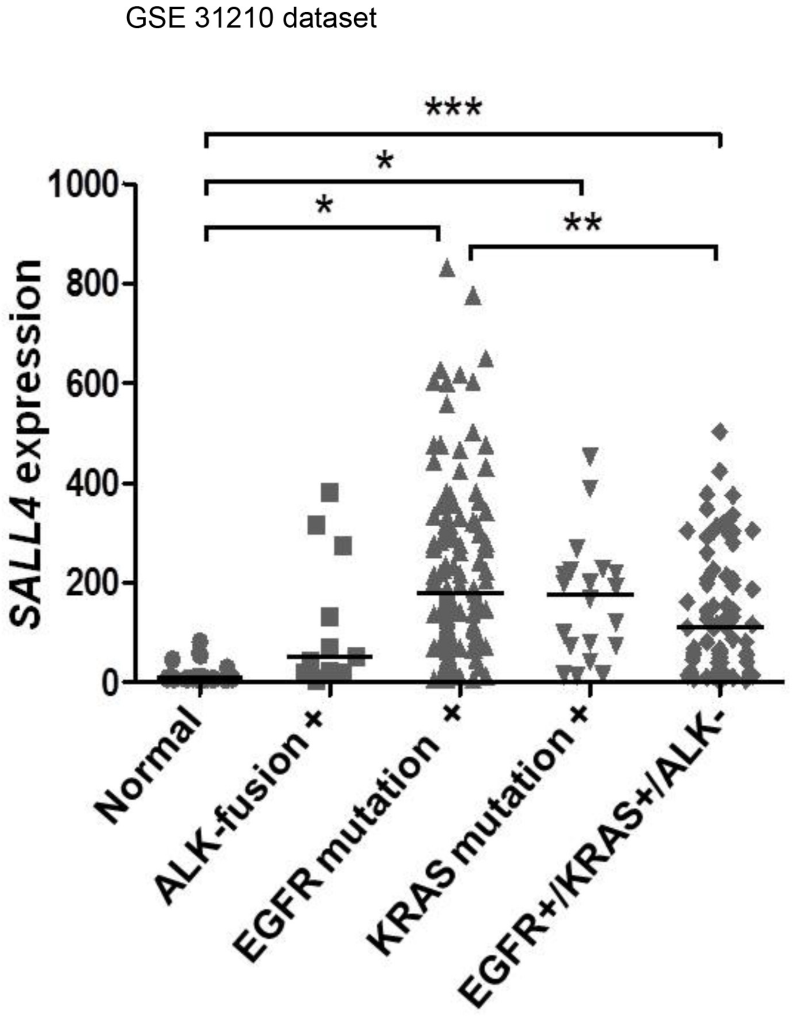

Supplementary Figure S2: *SALL4* mRNA expression in lung cancer patients with various genomic abnormalities. Primary human lung cancer samples were extracted from the GEO database (Accession No. GSE31210) and categorized into different groups based on their genomic aberrations, including ALK fusion +, EGFR mutation +, KRAS mutation +, and EGFR mutation +/KRAS mutation +/ ALK -. Higher *SALL4* expression is observed in samples with EGFR and KRAS mutations, but not in ALK fusion samples.* $P < 0.05$, *** $P < 0.001$.

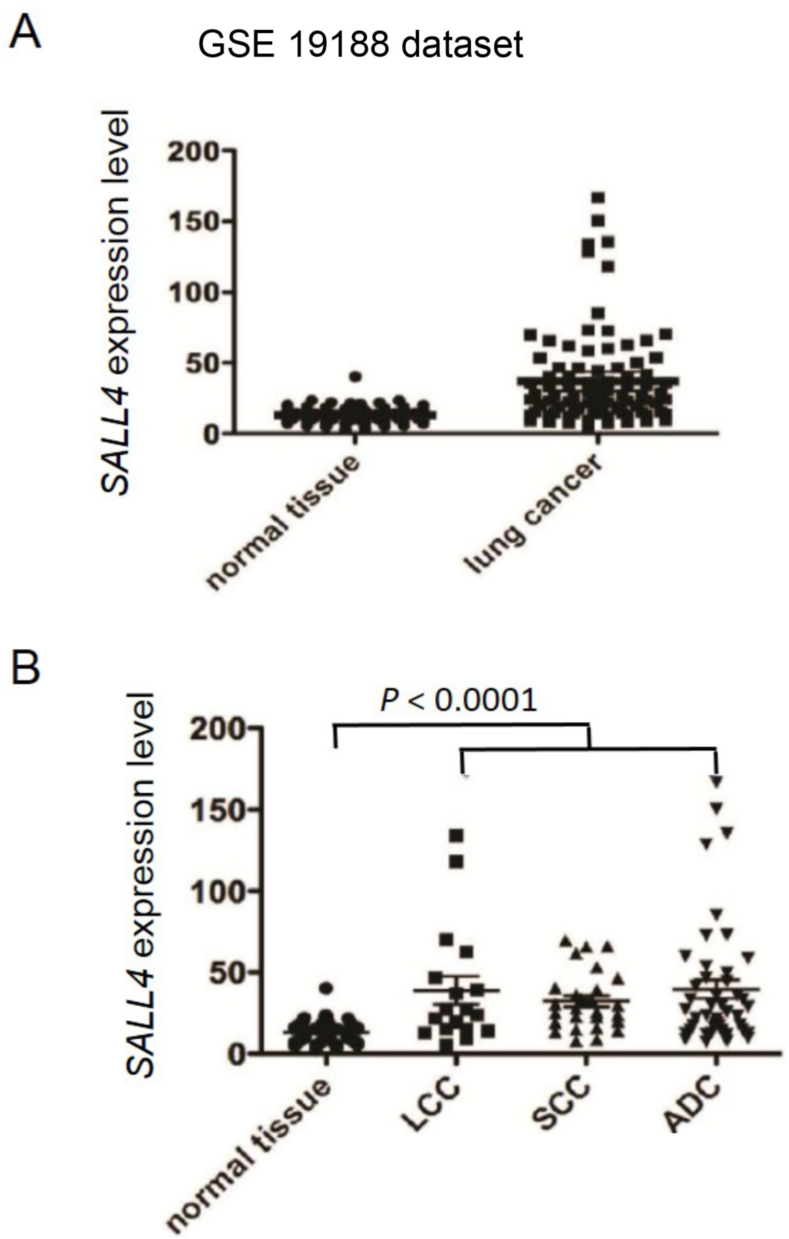

Supplementary Figure S3: *SALL4* expression is elevated in primary lung cancers. a. Increased *SALL4* mRNA expression is observed in lung cancer patients as compared to the normal tissues, from data extracted from the GEO database (Accession No. GSE19188). **b.** *SALL4* mRNA expression in different subtypes of NSCLCs. LCC = large cell carcinoma; SCC = squamous cell carcinoma; ADC = adenocarcinoma. (*Continued*)

C

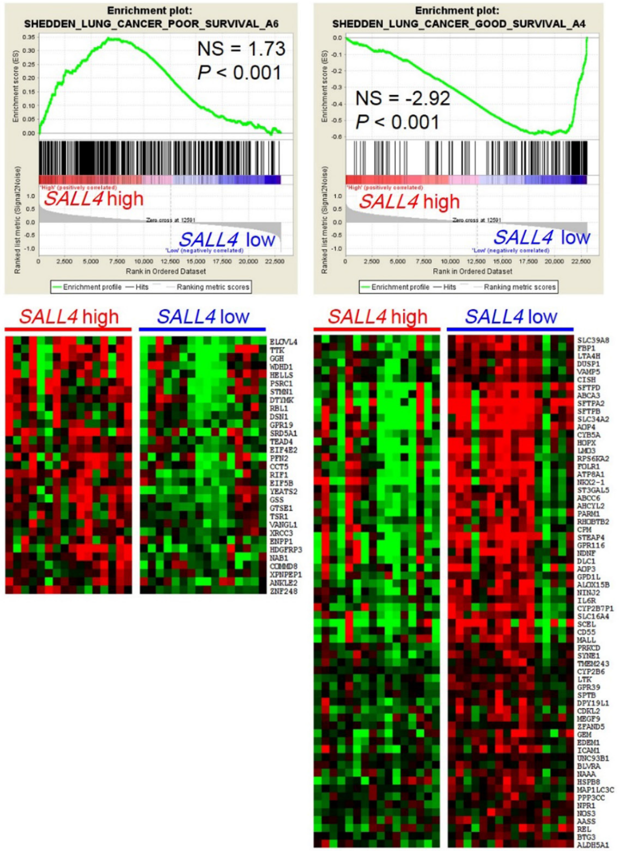

TCGA lung adenocarcinoma dataset

D

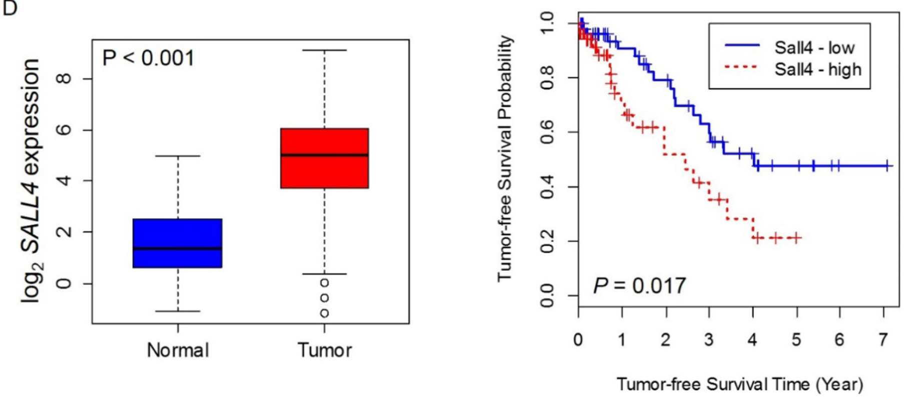

Supplementary Figure S3: (*Continued*) *SALL4* expression is elevated in primary lung cancers. c. GSEA analysis shows enrichment of poor survival signature in lung cancers with high *SALL4* expression, and enrichment of good survival signature in lung cancers with low *SALL4* expression. **d.** Using another lung adenocarcinoma dataset from TCGA, significantly increased *SALL4* mRNA expression is also observed in lung cancer patients as compared to matched normal tissues (left). Kaplan Meier analysis demonstrated poorer survival advantage for patients with upregulated *SALL4* expression (right).

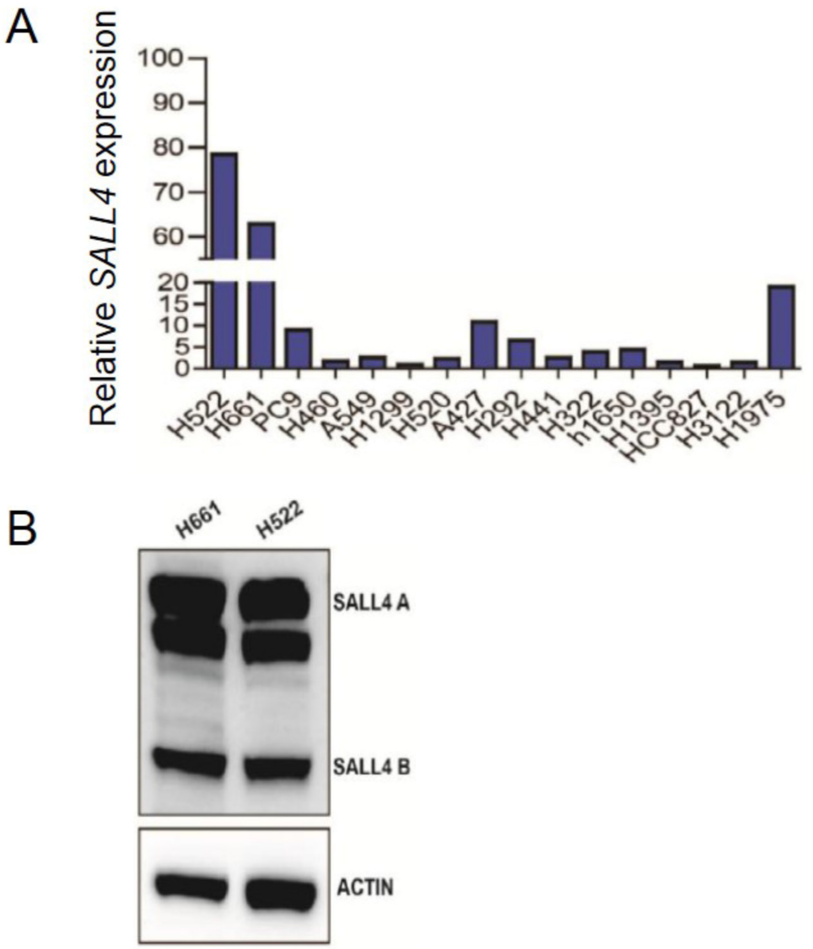

Supplementary Figure S4: Endogenous SALL4 mRNA and protein expression in lung cancer cell lines. a. qPCR data shows endogenous *SALL4* mRNA expression in a panel of lung cancer cell lines. **b.** Western blot shows high SALL4 expression in H661 and H522 cell lines.

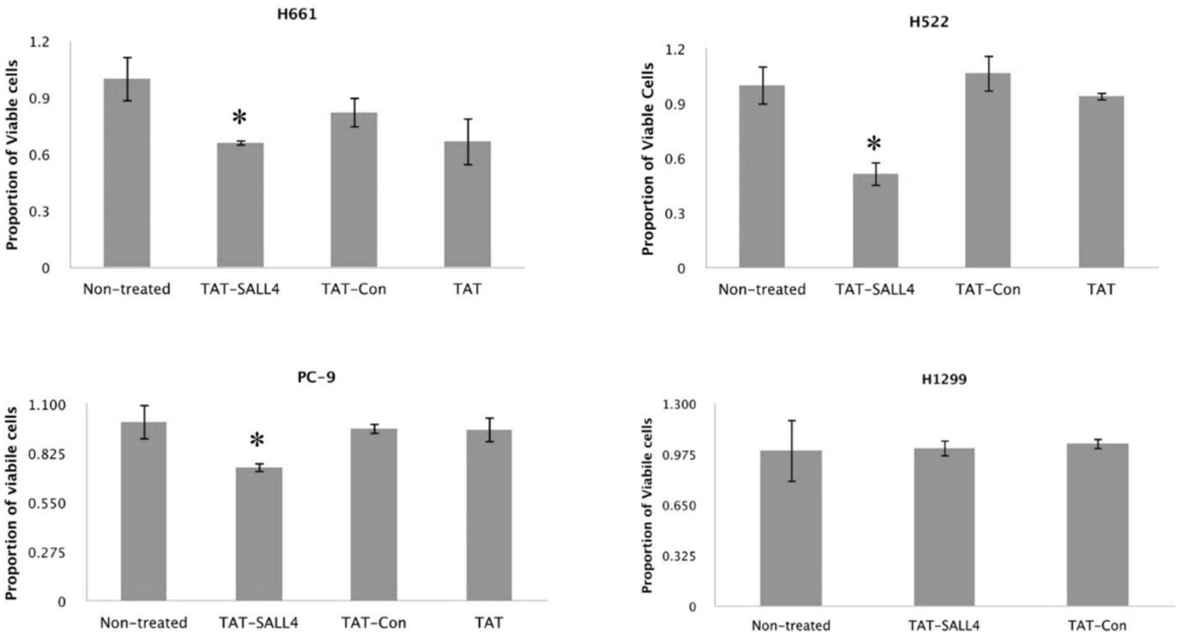

Supplementary Figure S5: Functional inhibition of SALL4 by a specific peptide inhibitor led to decreased cell viability of lung cancer cells. Cellular viability of lung cancer cell lines (H661, H522, PC-9, and H1299) 72 hours after treatments with TAT, TAT- SALL4, and TAT-control (TAT-con) peptides was evaluated by the MTT assay. Data were normalized to the non-treated control, and represent the mean values (± s.d.) of quadruplicate cultures from two independent experiments. SALL4 functional inhibition by TAT-SALL4 peptide significantly reduced cell viability in SALL4 positive lung cancer lines (H661, H522, and PC-9), but not in the SALL4 negative lung cancer line H1299. In contrast, treatment with TAT-con peptide or TAT alone only led to a slight decreased in cell viability in these cell lines. $^{*}P < 0.05$.

Supplementary Table S1: SALL4 gene signature generated from GSE31210 dataset by comparing samples with high vs. low *SALL4* expression (black indicates upregulated genes; red indicates downregulated genes).

See Supplementary File 1

Supplementary Table S2: SALL4 gene signature generated from GSE19188 dataset by comparing samples with high vs. low *SALL4* expression (black indicates upregulated genes; red indicates downregulated genes).

See Supplementary File 2

Supplementary Table S3: SALL4 gene signature generated from SALL4 knocked down H661 lung cancer cell line (black indicates upregulated genes; red indicates downregulated genes).

See Supplementary File 3

Supplementary Table S4: Drug list obtained from connectivity map analysis

SALL4 KD *vs* patient sample 1	SALL4 KD vs patient samples 2	Common list in three datasets
Azacitidine	Resveratrol	Resveratrol
Resveratrol	Thioridazine	Daunorubicin
0175029-0000	Doxylamine	Entinostat (MS-275)
Alsterpaullone	Fluphenazine	
Loracarbef	Camptothecin	
Daunorubicin	Daunorubicin	
Entinostat (MS-275)	Suloctidil	
Ellipticine	Entinostat (MS-275)	
Naltrexone	Staurosporine	
Prestwick-692	Prestwick-984	
Aminophylline	Blebbistatin	
Lanatoside C	7-aminocephalosporanic acid	
Ethosuximide	Sulconazole	
Isoconazole	Chenodeoxycholic acid	
Clorsulon	Perhexiline	
Nitrofural	Dipivefrine	
Gliquidone	Pyrimethamine	
	Felbinac	
	Amoxapine	
	Tropicamide	
	2-aminobenzenesulfonamide	
	Fluorometholone	
	Protriptyline	
	Ciclopirox	
	Ciprofibrate	
	Gentamicin	
	STOCK1N-35696	
	Piretanide	
