# Supplementary material for: Targeting SALL4 by entinostat in lung cancer
Source: Oncotarget. 2016 Sep 26;7(46):75425–40. doi: 10.18632/oncotarget.12251 (PMC5342750; doi:10.18632/oncotarget.12251)
Supplement: Supplementary file 2 [file oncotarget-07-75425-s002.docx]

**Supplementary Table 1.** SALL4 gene signature generated from GSE31210 dataset by comparing samples with high vs. low *SALL4* expression (black indicates upregulated genes; red indicates downregulated genes).

| Probe ID | Gene name |
| --- | --- |
| 221678_at | ABHD6 |
| 220676_at | ADAMTS8 |
| 203143_s_at | KIAA0040 |
| 204482_at | CLDN5 |
| 203543_s_at | KLF9 |
| 213914_s_at | SPTBN1 |
| 209481_at | SNRK |
| 218711_s_at | SDPR |
| 202112_at | VWF |
| 205846_at | PTPRB |
| 200671_s_at | SPTBN1 |
| 203144_s_at | KIAA0040 |
| 202877_s_at | CD93 |
| 205471_s_at | DACH1 |
| 204396_s_at | GRK5 |
| 200672_x_at | SPTBN1 |
| 202746_at | ITM2A |
| 201348_at | GPX3 |
| 217781_s_at | ZNF106 |
| 203980_at | FABP4 |
| 215918_s_at | SPTBN1 |
| 220518_at | ABI3BP |
| 45288_at | ABHD6 |
| 206481_s_at | LDB2 |
| 202878_s_at | CD93 |
| 209612_s_at | ADH1B |
| 205382_s_at | CFD |
| 209613_s_at | ADH1B |
| 204683_at | ICAM2 |
| 221841_s_at | KLF4 |
| 220170_at | FHL5 |
| 220266_s_at | KLF4 |
| 210190_at | STX11 |
| 221679_s_at | ABHD6 |
| 219761_at | CLEC1A |
| 206868_at | STARD8 |
| 207474_at | SNRK |
| 204731_at | TGFBR3 |
| 203542_s_at | KLF9 |
| 204395_s_at | GRK5 |
| 213974_at | ADAMTSL3 |
| 214091_s_at | GPX3 |
| 205960_at | PDK4 |
| 219436_s_at | EMCN |
| 219719_at | HIGD1B |
| 203541_s_at | KLF9 |
| 202747_s_at | ITM2A |
| 205472_s_at | DACH1 |
| 220471_s_at | MYCT1 |
| 213620_s_at | ICAM2 |
| 209614_at | ADH1B |
| 206030_at | ASPA |
| 217177_s_at | PTPRB |
| 220677_s_at | ADAMTS8 |
| 204719_at | ABCA8 |
| 221552_at | ABHD6 |
| 212071_s_at | SPTBN1 |
| 204883_s_at | HUS1 |
| 203878_s_at | MMP11 |
| 205941_s_at | COL10A1 |
| 219623_at | ACTR5 |
| 212353_at | SULF1 |
| 204884_s_at | HUS1 |
| 207625_s_at | CBFA2T2 |
| 203956_at | MORC2 |
| 209145_s_at | CBFA2T2 |
| 220002_at | KIF26B |
| 217428_s_at | COL10A1 |
| 212354_at | SULF1 |
| 204320_at | COL11A1 |
| 203083_at | THBS2 |
| 216242_x_at | POLR2J2 /// POLR2J3 /// POLR2J4 /// UPK3BL |
| 206224_at | CST1 |
| 217618_x_at | HUS1 |
| 221900_at | COL8A2 |
| 219773_at | NOX4 |
| 219655_at | C7orf10 |
| 210165_at | DNASE1 |
| 220661_s_at | ZNF692 |
| 209144_s_at | CBFA2T2 |
| 222147_s_at | ACTR5 |
| 201870_at | TOMM34 |
| 216863_s_at | MORC2 |
| 52651_at | COL8A2 |
| 221780_s_at | DDX27 |
| 215693_x_at | DDX27 |
| 206994_at | CST4 |
| 212344_at | SULF1 |
| 206547_s_at | PPEF1 |
| 214740_at | POLR2J2 /// POLR2J3 /// POLR2J4 /// UPK3BL |
| 37892_at | COL11A1 |
| 203876_s_at | MMP11 |
