# Supplementary material for: Targeting SALL4 by entinostat in lung cancer
Source: Oncotarget. 2016 Sep 26;7(46):75425–40. doi: 10.18632/oncotarget.12251 (PMC5342750; doi:10.18632/oncotarget.12251)
Supplement: Supplementary file 3 [file oncotarget-07-75425-s003.docx]

**Supplementary Table 2.** SALL4 gene signature generated from GSE19188 dataset by comparing samples with high vs. low *SALL4* expression (black indicates upregulated genes; red indicates downregulated genes)

| Probe ID | Gene name |
| --- | --- |
| 203549_s_at | LPL |
| 207542_s_at | AQP1 |
| 206049_at | SELP |
| 209613_s_at | ADH1B |
| 219777_at | GIMAP6 |
| 208335_s_at | DARC |
| 205789_at | CD1D |
| 219584_at | PLA1A |
| 219243_at | GIMAP4 |
| 214470_at | KLRB1 |
| 201988_s_at | CREBL2 |
| 203548_s_at | LPL |
| 203799_at | CD302 /// LY75-CD302 |
| 219079_at | CYB5R4 |
| 204683_at | ICAM2 |
| 209276_s_at | GLRX |
| 34210_at | CD52 |
| 209614_at | ADH1B |
| 210066_s_at | AQP4 |
| 206311_s_at | PLA2G1B |
| 213620_s_at | ICAM2 |
| 207794_at | CCR2 |
| 206662_at | GLRX |
| 213293_s_at | TRIM22 |
| 209696_at | FBP1 |
| 220380_at | DNASE2B |
| 213115_at | ATG4A |
| 203378_at | PCF11 |
| 210067_at | AQP4 |
| 201990_s_at | CREBL2 |
| 219505_at | CECR1 |
| 205821_at | KLRC4-KLRK1 /// KLRK1 |
| 205504_at | BTK |
| 206978_at | CCR2 |
| 210068_s_at | AQP4 |
| 208771_s_at | LTA4H |
| 201989_s_at | CREBL2 |
| 209047_at | AQP1 |
| 218065_s_at | TMEM9B |
| 204661_at | CD52 |
| 209612_s_at | ADH1B |
| 221887_s_at | DFNB31 |
| 220632_s_at | POMT2 |
| 211928_at | DYNC1H1 |
| 203627_at | IGF1R |
| 206858_s_at | HOXC6 |
| 207115_x_at | MBTD1 |
| 214872_at | RIF1 |
| 215468_at | LOC647070 |
| 219269_at | HMBOX1 |
| 220661_s_at | ZNF692 |
| 205598_at | TRAIP |
| 208151_x_at | DDX17 |
| 207011_s_at | PTK7 |
| 204140_at | TPST1 |
| 203628_at | IGF1R |
| 50277_at | GGA1 |
| 218682_s_at | SLC4A1AP |
| 208441_at | IGF1R |
| 217560_at | GGA1 |
| 210005_at | GART |
| 47553_at | DFNB31 |
