# Supplementary material for: Targeting SALL4 by entinostat in lung cancer
Source: Oncotarget. 2016 Sep 26;7(46):75425–40. doi: 10.18632/oncotarget.12251 (PMC5342750; doi:10.18632/oncotarget.12251)
Supplement: Supplementary file 4 [file oncotarget-07-75425-s004.docx]

**Supplementary Table 3.** SALL4 gene signature generated from SALL4 knocked down H661 lung cancer cell line (black indicates upregulated genes; red indicates downregulated genes)

| Probe ID | Gene name |
| --- | --- |
| 204333_s_at | AGA |
| 213446_s_at | IQGAP1 |
| 212136_at | ATP2B4 |
| 221759_at | G6PC3 |
| 1405_i_at | CCL5 |
| 218986_s_at | DDX60 |
| 202868_s_at | POP4 |
| 218943_s_at | DDX58 |
| 203153_at | IFIT1 |
| 214072_x_at | NENF |
| 213969_x_at | RPL29 |
| 201009_s_at | LOC101060503 /// TXNIP |
| 210797_s_at | OASL |
| 213673_x_at | NENF |
| 212135_s_at | ATP2B4 |
| 219863_at | HERC5 |
| 217502_at | IFIT2 |
| 208864_s_at | TXN |
| 205660_at | OASL |
| 216020_at | IFIH1 |
| 214075_at | NENF |
| 214059_at | IFI44 |
| 208736_at | ARPC3 |
| 222212_s_at | CERS2 |
| 216064_s_at | AGA |
| 44654_at | G6PC3 |
| 200791_s_at | IQGAP1 |
| 219209_at | IFIH1 |
| 207038_at | SLC16A6 |
| 217874_at | SUCLG1 |
| 218406_x_at | NENF |
| 201010_s_at | LOC101060503 /// TXNIP |
| 210840_s_at | IQGAP1 |
| 218407_x_at | NENF |
| 204751_x_at | DSC2 |
| 204332_s_at | AGA |
| 201008_s_at | LOC101060503 /// TXNIP |
| 221437_s_at | MRPS15 |
| 216609_at | TXN |
| 204750_s_at | DSC2 |
| 205410_s_at | ATP2B4 |
| 209208_at | MPDU1 |
| 214125_s_at | NENF |
| 200823_x_at | RPL29 |
| 204655_at | CCL5 |
| 204439_at | IFI44L |
| 204747_at | IFIT3 |
| 204533_at | CXCL10 |
| 208289_s_at | EI24 |
| 208066_s_at | GTF2B |
| 205063_at | GEMIN2 |
| 218346_s_at | SESN1 |
| 211114_x_at | GEMIN2 |
| 209069_s_at | H3F3A /// H3F3B /// MIR4738 |
| 209778_at | TRIP11 |
| 206667_s_at | SCAMP1 |
| 221703_at | BRIP1 |
| 214834_at | PAR5 |
| 214724_at | DIXDC1 |
| 211929_at | HNRNPA3 |
| 211930_at | HNRNPA3 |
| 211997_x_at | H3F3A /// H3F3B /// MIR4738 |
| 211931_s_at | HNRNPA3 /// HNRNPA3P1 |
| 210760_x_at | TRIP11 |
| 214938_x_at | HMGB1 |
| 200679_x_at | HMGB1 |
| 211115_x_at | GEMIN2 |
| 206668_s_at | SCAMP1 |
| 209585_s_at | MINPP1 |
| 212479_s_at | RMND5A |
| 220940_at | ANKRD36B |
| 216396_s_at | EI24 |
| 212425_at | SCAMP1 |
| 211933_s_at | HNRNPA3 /// HNRNPA3P1 |
| 211999_at | H3F3A /// H3F3B /// MIR4738 |
| 222204_s_at | RRN3 |
| 212478_at | RMND5A |
| 212482_at | RMND5A |
| 218604_at | LEMD3 |
| 211998_at | H3F3A /// H3F3B /// MIR4738 |
| 220730_at | LOC642533 |
| 200680_x_at | HMGB1 |
| 218932_at | ZNHIT6 |
| 211932_at | HNRNPA3 |
| 210779_x_at | GEMIN2 |
| 219006_at | NDUFAF4 |
